# Meta-Analysis of Diagnostic Performance of Instantaneous Wave-Free Ratio versus Quantitative Flow Ratio for Detecting the Functional Significance of Coronary Stenosis

**DOI:** 10.1155/2019/5828931

**Published:** 2019-04-18

**Authors:** Wenjie Zuo, Mingming Yang, Yifan Chen, Aiming Xie, Lijuan Chen, Genshan Ma

**Affiliations:** Department of Cardiology, Zhongda Hospital, School of Medicine, Southeast University, Nanjing 210009, China

## Abstract

**Background:**

Fractional flow reserve (FFR), as a functional measurement of coronary stenosis, is recommended for guiding revascularization in intermediate coronary lesions. However, it still remains underutilized for potential reasons including time consumption, costs, or contraindications associated with adenosine administration. Here we performed this meta-analysis to assess the diagnostic performance of two adenosine-free indices, instantaneous wave free-ratio (iFR), and quantitative flow ratio (QFR) in evaluating coronary stenosis severity with FFR as the reference standard.

**Methods:**

PubMed, Embase, and Cochrane Central Register of Controlled Trials (CENTRAL) were searched to include relevant studies with the diagnostic accuracy of iFR or QFR referenced to FFR. A bivariate model was applied to pool diagnostic parameters. We used Cochran's Q test and I^2^ index to assess heterogeneity and identify the potential source of heterogeneity by meta-regression.

**Results:**

A total of 8213 lesions from 28 studies (19 for iFR and 9 for QFR) were included in this meta-analysis. The pooled sensitivity and specificity were 0.79 (95% CI, 0.75 to 0.83) and 0.85 (95% CI, 0.82 to 0.87) for iFR and 0.90 (95% CI, 0.84 to 0.93) and 0.88 (95% CI, 0.86 to 0.90) for QFR, respectively. Significantly higher sensitivity and specificity were observed in the bivariate analysis for QFR than for iFR (P < 0.001 for both). The area under summary receiver-operating curve of iFR and QFR was 0.89 (95% CI, 0.86 to 0.92) and 0.92 (95% CI, 0.89 to 0.94).

**Conclusion:**

Evidence suggests that both of the two indices have good performance in detecting functional ischemia of coronary arteries and QFR might be a promising method without requiring the pressure wire. Further application of QFR may potentially provide important information to clinicians in the assessment of coronary lesions.

## 1. Introduction

Fractional flow reserve (FFR) has emerged as an essential physiological index in the assessment of coronary artery stenosis since its introduction more than 2 decades ago [1]. Routine FFR measurement was shown to be superior to angiography-guided revascularization with a significant reduction of mortality and myocardial infarction at 2 years [2]. The 2014 ESC/EACTS guideline recommended FFR to assess the severity of lesions in stable patients without available evidence of ischemia (class I, level of evidence A) [3]. However, there are still limitations for FFR in clinical practice including additional time consumption, costs, and vasodilator administration [4]. Instantaneous wave-free ratio (iFR) and quantitative flow ratio (QFR) were developed to be alternatives to FFR in the assessment of coronary stenosis severity [5, 6]. Without the requirement of hyperemic agents, iFR is obtained in a specific time in diastole called wave-free period when there is minimal and constant resistance [5]. QFR, a novel method in physiological assessment of stenosis, is the computational FFR based on 3-dimensional angiographic reconstruction without pharmacologically induced hyperemia [6, 7]. Recently, it has been shown that iFR-guided revascularization could be noninferior to the interventional strategy guided by FFR with respect to the 1-year major adverse cardiac events according to the DEFINE-FLAIR (Functional Lesion Assessment of Intermediate Stenosis to Guide Revascularization) and iFR SWEDEHEART (The Instantaneous Wave-free Ratio versus Fractional Flow Reserve in Patients with Stable Angina Pectoris or Acute Coronary Syndrome) trials [8, 9]. The FAVOR (Functional Diagnostic Accuracy of Quantitative Flow Ratio in Online Assessment of Coronary Stenosis) II CHINA study demonstrated high diagnostic accuracy of QFR [7]. However, previous two meta-analyses may be underpowered due to limited size and there is no systematic comparison between iFR and QFR to evaluate their diagnostic performance [10, 11]. Compared with the solid evidence of FFR, further investigations are warranted to unveil the uncertainty of the two novel indices. We therefore performed this meta-analysis to update information and exclude studies with duplicated data for a better understanding of iFR and QFR in assessing coronary stenosis severity.

## 2. Methods

This meta-analysis was conducted according to the Preferred Reporting Items for Systematic Reviews and Meta-Analyses (PRISMA) statement [12].

### 2.1. Search Strategy

PubMed, Embase, and Cochrane Central Register of Controlled Trials (CENTRAL) were searched to retrieve relevant records to evaluate the diagnostic accuracy of iFR or QFR with reference to FFR from inception to 11 July 2018 and there is no restriction of language. We used a combination of MeSH/Emtree and entry terms of iFR/QFR and FFR to perform a comprehensive search. The search was limited to literature published as full-text articles on peer-reviewed journals. Conference abstracts were excluded due to limited data and potential of bias. In the search process of Embase, the publication types were limited as “article” and “article in press” to exclude reviews, editorials, or conference abstracts. The details of the search strategy are shown in Supplementary materials.

### 2.2. Study Selection

Our inclusion criteria were as follows: (1) the accuracy of iFR or QFR was assessed with FFR as a reference; (2) sufficient data must be provided to allow us to calculate the number of true positives, false negatives, false positives, and true negatives, which construct the 2×2 contingency table. Studies were excluded if they use data that previous studies have reported or with insufficient data. Electronic records were screened independently by two authors and any discrepancy was resolved by a third investigator.

### 2.3. Data Extraction and Quality Assessment

Data extraction and quality assessment were conducted independently by two investigators. The following information of included studies was collected: the first author, publication year, study type, inclusion and exclusion criteria, cutoffs of iFR/QFR and FFR, general demographics, characteristics of lesions, and diagnostic parameters.

The risk of bias was assessed using Review Manager 5.3 (Nordic Cochrane Centre, The Cochrane Collaboration, 2014, Copenhagen, Denmark) with the Quality Assessment of Diagnostic Accuracy Studies 2 (QUADAS-2) [13] by two investigators. It consists of four key components: patient selection, index test, reference standard, flow, and timing with a list of 11 signaling questions (yes, no, or unclear) to assist in judgments about the risk of bias. Each component is assessed in terms of risk of bias and the first three include concerns regarding applicability.

### 2.4. Statistical Analysis

True positives, false negatives, false positives, and true negatives were calculated from the reported data including sensitivity, specificity, positive and negative likelihood ratios (LR+ and LR-), and sample size. A bivariate mixed-effects regression model was applied to pool diagnostic parameters. Cochran's Q test and I^2^ index were applied to assess potential heterogeneity. Significant heterogeneity was considered to exist among studies when P < 0.1 or I^2^ > 50%. The source of heterogeneity was identified by meta-regression analysis. The logit of sensitivity and (1-specificity) were used to estimate the Spearman correlation coefficient to investigate the diagnostic threshold effect. Bivariate comparison of sensitivity and specificity between physiological indices (iFR and QFR) was conducted in the model described by Reitsma et al. [14]. The physiological index was added as a covariate to our bivariate model to observe the potential diagnostic difference between iFR and QFR. The logit estimates of sensitivity, specificity, and respective variances were used to draw a summary receiver-operating characteristic (ROC) curve. To investigate publication bias, Deek's funnel plot asymmetry test was performed and significant asymmetry was indicated when the P value is less than 0.05. Statistical analysis was performed using the MIDAS module for STATA, version 14 (StataCorp, College Station, Texas, USA) with a two-tailed P value (defined statistical significance when P < 0.05).

## 3. Results

### 3.1. Characteristics of Included Studies

**Figure 1:**
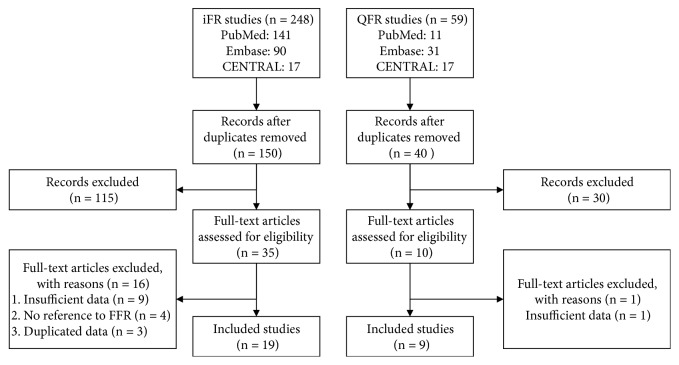
Flow diagram of search and study selection.

We screened 307 electronic records (248 for iFR and 59 for QFR) based on titles and abstracts. A total of 262 pieces of literature were excluded because they are duplications or conference abstracts. After carefully reviewing the full texts, we excluded 16 iFR and 1 QFR studies based on the exclusion criteria. There were 19 iFR and 9 QFR studies which met the inclusion criteria retrieved in our final analysis (Figure 1). Overall, our meta-analysis consists of 19 studies (6492 lesions) and 9 studies (1721 lesions) for iFR and QFR, respectively. It was noted that a retrospective, single-center trial [15] involving 100 patients is the only study comparing iFR and QFR directly, which was included in both the iFR group and the QFR group. The details of 28 studies are described in Table 1 including the first author, published year, the number of lesions, research type, FFR cutoff, and iFR/QFR cutoff. Baseline characteristics of patients and vessels are shown in Tables 2 and 3, respectively. Inclusion and exclusion criteria of studies can be found in Table S1.

Most studies of iFR (13 of 19) were performed at a single site and only 6 are multicenter studies [16–21]. Of these single-center studies, nearly 70% trials (9 of 13) were conducted in Europe while 3 in Japan [22–24] and 1 in Canada [25]. The number of lesions ranged from 40 to 1593 with a median of 229. Only 36.8% of studies (7 of 19) [15–17, 21–23, 26] have a clear statement of blinded strategy. The cutoffs of iFR did not vary greatly between diagnostic studies, with a range from 0.86 to 0.92 while 0.90 is adopted in most studies (7 of 19) [16–18, 21, 25, 27, 28]. The cutoff of FFR is 0.80 for all studies.

Four of nine studies [15, 29–31] of QFR were single-site studies while others involved multiple hospitals. More than half of studies (5 of 9) [6, 31–34] included centers in Europe. The number of included vessels ranged from 49 to 330 (median, 151 vessels). The description of applying a blinded strategy was provided except for 1 study [30]. All studies adopted 0.80 as the cutoff of FFR and QFR.

### 3.2. Diagnostic Performance of iFR and QFR

**Figure 2:**
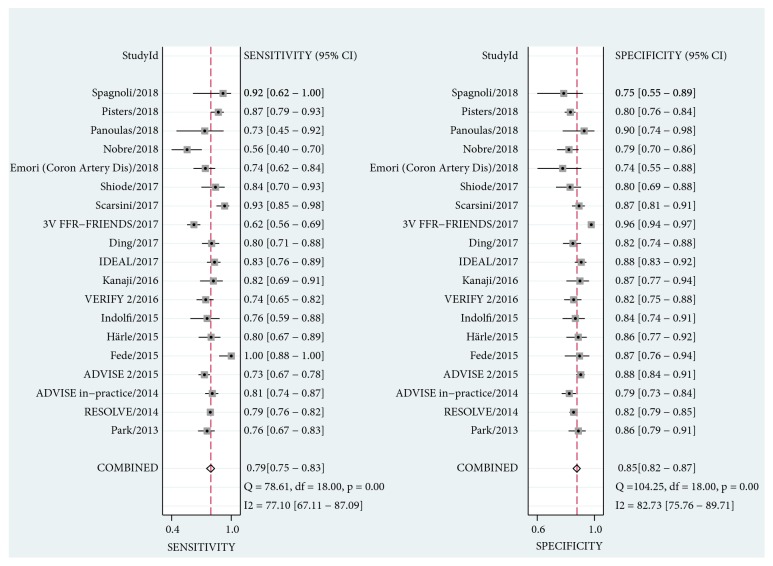
Forest plots for sensitivity and specificity of iFR. CI, confidence intervals.

**Figure 3:**
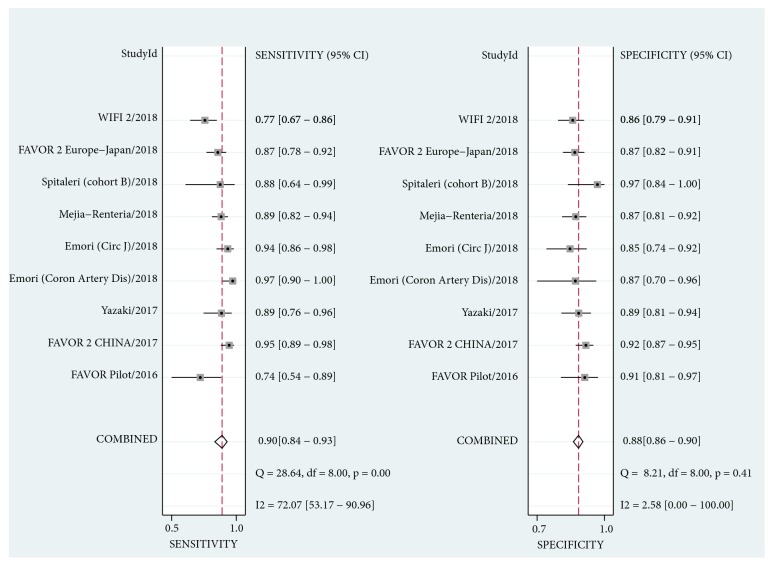
Forest plots for sensitivity and specificity of QFR. CI, confidence intervals.

**Figure 4:**
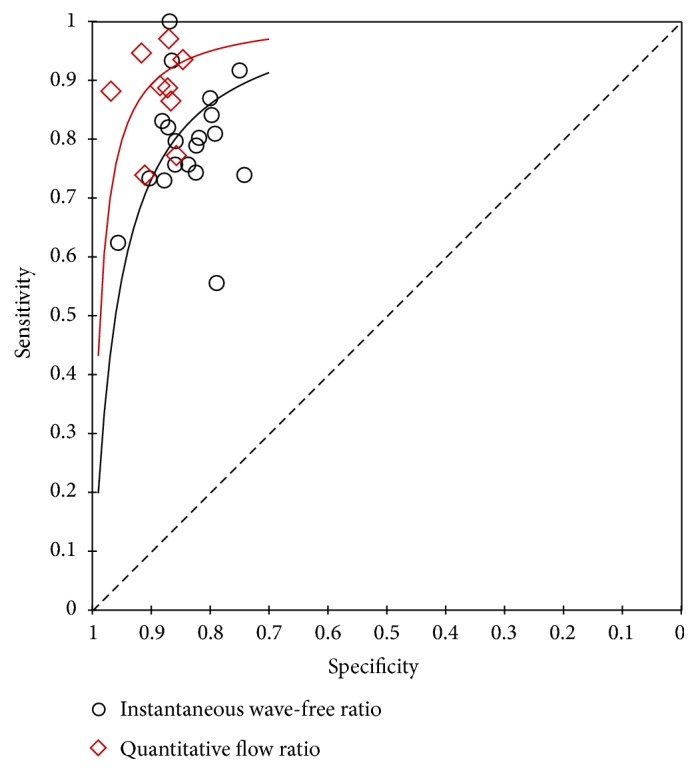
Summary receiver-operating characteristic curve for iFR and QFR.

As shown in Figure 2, the pooled sensitivity and specificity were 0.79 (95% CI, 0.75 to 0.83) and 0.85 (95% CI, 0.82 to 0.87) for iFR. The estimate of LR+, LR-, and diagnostic odds ratio was 5.3 (95% CI, 4.4 to 6.3), 0.24 (95% CI, 0.20 to 0.29), and 22 (95% CI, 17 to 29), respectively. For QFR, the pooled diagnostic parameters were sensitivity, 0.90 (95% CI, 0.84 to 0.93); specificity, 0.88 (95% CI, 0.86 to 0.90); LR+, 7.8 (95% CI, 6.3 to 9.6); LR-, 0.12 (95% CI, 0.08 to 0.18); diagnostic odds ratio, 66 (95% CI, 38 to 116) (Figure 3). Significant difference was observed in sensitivity and specificity (P < 0.001 for both) for both iFR and QFR when we investigated the potential effect of covariate (physiological methods) on the bivariate model, indicating the superiority of QFR to iFR. The summary ROC curves of iFR and QFR was shown in Figure 4. The area under the curve (AUC) was 0.89 (95% CI, 0.86 to 0.92) for iFR and 0.92 (95% CI, 0.89 to 0.94) for QFR. However, significant heterogeneity was found between studies for pooled sensitivity (I^2^ = 77.10%, P < 0.01), specificity (I^2^ = 82.73%, P < 0.01) of iFR, and sensitivity of QFR (I^2^ = 72.07%, P < 0.01). For iFR, the correlation coefficient was -0.40 and the proportion of heterogeneity due to threshold effect was 0.16, suggesting no evidence of a threshold effect.

### 3.3. Meta-Regression Analysis

To identify the sources of significant heterogeneity, we performed meta-regression analyses and four factors were defined as covariates: number of centers (single or multiple), blinded strategy, sample size (number of lesions), and study design (prospective or retrospective). The results indicated that the number of centers, blinded strategy, and study design might be significant factors while the sample size did not have a remarkable effect on the heterogeneity for sensitivity and specificity of iFR. Interestingly, a similar result was obtained from the analysis of QFR except that blinded strategy had no effect on heterogeneity for sensitivity.

### 3.4. Quality Assessment and Publication Bias

The methodological quality of iFR and FFR studies was summarized in Figures S1 and S2, respectively. The overall quality of iFR studies varied from moderate to high. The low risk of bias was achieved by more than 70% of studies in four areas including patient selection, index test, reference standard, flow, and timing. For the index test, only one study [28] had a high risk of bias due to lack of the blinded strategy and a prespecified threshold. For applicability concerns, low concerns were fulfilled by all studies except two [25, 28]. Interestingly, the QFR studies had higher quality than the iFR studies. The unclear risk of bias was obtained in only 4 studies [6, 29, 30, 32]. All studies obtained low concerns regarding applicability for patient selection, index test, and reference standard. As shown in Figures S3 and S4, there is no evident publication bias for both iFR (P = 0.55) and QFR (P = 0.65) according to Deek's asymmetry test.

## 4. Discussion

This is the first meta-analysis comparing the diagnostic performance of iFR and QFR with conventional FFR as the gold standard. In this study, both techniques exhibit inspiring diagnostic performance referenced to FFR. Our results show that, compared with iFR, QFR has better sensitivity and specificity in detecting the functional ischemia of coronary arteries, which is consistent with the results of a recent head-to-head comparison [15]. Furthermore, QFR is superior to iFR with higher diagnostic odds ratio and AUC. These findings thus lend support to QFR as a noninvasive diagnostic method that can accurately detect functionally significant coronary stenosis.

The physiological assessment was used to evaluate the coronary stenosis severity in addition to coronary angiography since the emergence of FFR in 1993 [1]. The application of physiological measurement provided interventional cardiologists with the ability to accurately determine the severity of stenosis [35]. It has been demonstrated that FFR-guided percutaneous intervention (PCI) with optimal medical therapy is superior to optimal medical therapy alone [36]. Nevertheless, the use of FFR in catheter laboratories was limited due to the cost and contraindications associated with adenosine [4].

Two novel physiological methods, iFR and QFR, have been recently introduced to accurately detect functionally significant coronary lesions [5, 6]. In a specific period in diastole known as the wave-free period, iFR is calculated from the ratio of distal coronary pressure (Pd) to proximal aortic pressure (Pa). There is a proportional relationship between intracoronary pressure and flow velocity and waves are quiescent during such a period [4, 5]. Therefore, iFR would achieve the measurement of coronary lesions without the requirement of a hyperemic agent. In contrast to iFR, QFR is an angiography-based method to estimate FFR without using pressure wires. It is automatically calculated in the online system using two angiographic images acquired at different angles ≥ 25° and a frame count method to estimate contrast flow velocity [6, 7]. In the FAVOR II CHINA study, it was revealed that QFR had superior sensitivity (94.6% versus 62.5%) and specificity (91.7% versus 58.1%) to 2-dimensional quantitative coronary angiography (P <0.001 for both) [7]. This new diagnostic approach appears to be a promising technique for assessing intermediate coronary stenosis in the future when cost and time consumption are considered. The procedure time would also be shortened using an online system in the catheter laboratory with a median time of 5 minutes versus 7 minutes for FFR [33]. In addition, the computation of QFR can be achieved based on coronary angiography alone without another test using pressure wires or hyperemic agents [7].

As shown in Figures 2 and 3, our analysis showed an overall beneficial effect on the sensitivity and specificity of QFR versus iFR. Furthermore, this effect was confirmed to be statistically significant in sensitivity and specificity (P < 0.001 for both) when we regarded the diagnostic method as a covariate in our bivariate model. This benefit can also be observed in the summary ROC curves of iFR and QFR (AUC: 0.89 versus 0.92, respectively) (Figure 4). Our findings demonstrated that QFR has a high diagnostic performance with the pooled sensitivity of 0.90, the specificity of 0.88 and AUC of 0.92, which is consistent with previous studies [7, 33]. To the best of our knowledge, this is the first comprehensive review and comparison of iFR and QFR. Two previous meta-analyses assessed the diagnostic parameters of iFR for the evaluation of coronary stenosis [10, 11]. However, the reliability of these two analyses may be limited due to their small size. In addition, the RESOLVE study [17] had collected the data from ADVISE (Adenosine Vasodilator Independent Stenosis Evaluation) [5], VERIFY (Verification of Instantaneous Wave-Free Ratio and Fractional Flow Reserve for the Assessment of Coronary Artery Stenosis Severity in Everyday Practice) [37], and other studies. We thus excluded the ADVISE [5] and VERIFY [37] studies to reduce the potential risk of bias.

The results of this study indicate that as a promising novel tool, QFR can be applied in guiding coronary revascularization. Before the wide application of QFR in the catheter laboratory, solid evidence from randomized studies is required and two randomized controlled trials investigating clinical outcomes are in the recruiting process. FAVOR III Europe Japan Study (ClinicalTrials.gov Identifier: NCT03729739) is to investigate the noninferiority of QFR-based diagnostic strategy to a standard pressure wire guided strategy in terms of a composite endpoint of all-cause mortality, nonfatal myocardial infarction, and unplanned revascularization after 12 months [38]. This trial is planned to recruit participants from 33 hospitals across Europe and Japan and to randomize 2,000 patients 1:1 to either QFR- or FFR-guided strategy. The primary results are expected to be available in 2020. Similarly, the FAVOR III China study (ClinicalTrials.gov Identifier: NCT03656848) is also a prospective, randomized, multicenter trial but is to validate whether QFR-guided PCI is superior to angiography-guided PCI on clinical outcomes [39]. FAVOR III China study defines major adverse cardiac events (MACE) as a composite of all-cause mortality, myocardial infarction (MI), and any ischemia-driven revascularization at 1 year and results are also expected in 2020. The two studies will help us build a deep understanding of QFR-guided strategy on clinical outcomes.

Nevertheless, it is noted that applying physiological tests in clinical practice is more complex than simply evaluating diagnostic parameters. Discordance between iFR and FFR is common in patients with coronary intermediate lesions, especially for those lesions at the grey zone (FFR range: 0.75~0.80) [5, 17]. The numerical results obtained from the wave-free period may be affected by hemodynamic fluctuations, wire malposition, or drift [40]. It is necessary to repeat measurements when drifting ≥2 mm Hg because such a degree of drift may cause lesion misclassification in FFR, iFR, and whole-cycle Pd/Pa, especially when indices are near the cutoff value [41]. Present QFR calculation is derived from the 3-dimensional vessel reconstruction and a semiautomatic frame count method, which relies on the high-quality angiography and the manual correction by well-trained technicians [6, 7]. Experienced operators, appropriate projection angles, and a steady contrast dye flow are generally required to optimize angiographic quality. The use of QFR can avoid acute coronary events or dissection caused by crossing complex lesions with pressure wire [42]. However, it is also challenging to estimate functional significance for these complex lesions such as bifurcation, severe tortuosity, and ostial stenosis, warranting a more sophisticated algorithm for QFR analysis [7]. Reproducibility is another concern. Although previous studies [6, 33, 43] showed good interobserver reproducibility of QFR, further large-scale evidence is still needed. Taken together, advancements in these fields may translate current iFR or QFR analysis toward a user-friendly workflow to promote the wider application of functional measurements.

Future technical developments will provide us with multiple approaches to the severity of coronary stenosis. Different from isolating the wave-free period, the ratio of mean distal coronary pressure to mean aortic pressure (resting Pd/Pa) is measured in the whole cycle, which can be used as another alternative to FFR [4, 44]. A pooled analysis of 14 diagnostic studies indicated that the mean sensitivity and specificity of resting Pd/Pa were 0.77 and 0.82, respectively [45]. Kobayashi and his colleagues demonstrated an excellent agreement (AUC: 0.98) between resting Pd/Pa and iFR after evaluating 763 patients from 12 institutions [46]. Nevertheless, compared to resting Pd/Pa, the iFR was more sensitive to the difference in the severity of stenosis [44]. Similar to QFR, the virtual FFR derived from imagings including coronary computed tomography angiography (CCTA) [47], intravascular ultrasound (IVUS) [48], and optical coherence tomography (OCT) [49] combines the anatomical and physiological assessments without adverse effects from additional tests. Further results are awaited to determine the role of those techniques in detecting the physiological significance of coronary arteries.

There are certain limitations to this present study. First, we found fewer data associated with QFR because iFR was introduced earlier. More than 60% of the included studies (19 of 28) assessed the performance of iFR. Further information and ongoing head-to-head comparisons are needed to elucidate the relationship between these two methods. An ongoing clinical trial, DETECT ISCHEMIA (Determining the Functional Significance of Intermediate Stenoses in Ischemic Heart Disease; ClinicalTrials.gov Identifier: NCT03420131) was designed to investigate the diagnostic agreement between iFR and QFR in the setting of real world [50]. It is a prospective, observational, single-center study with 250 participants and estimated to be completed by October 2018. The results of this study may further elucidate the uncertainty of iFR and QFR and provide information about the revascularization guided by physiological measurements. Second, our results were heterogeneous. The meta-regression analysis indicated that several covariates (number of centers, blinded strategy, and study design) may explain the observed heterogeneity. Lastly, not all studies were designed prospectively. We did not exclude the retrospective analysis for a comprehensive review. However, significant heterogeneity was also observed in a previous meta-analysis [10] of iFR without retrospective studies, suggesting that the inclusion of retrospective studies may not be the main cause of heterogeneity.

In conclusion, our analysis confirms the impressive diagnostic performance of iFR and QFR for detecting the functional ischemia of coronary arteries. QFR might be considered as a reliable alternative to pressure wire-based iFR for its simplicity and better diagnostic accuracy. However, this superiority should be interpreted with caution due to observed heterogeneity, lack of randomized trials, and complex situations in clinical practice. Further randomized trials are warranted to unveil the value of QFR-based strategy in patients requiring functional evaluation.

## Figures and Tables

**Table 1:** Characteristics of included studies.

Included studies	Year	No. of lesions	Type	FFR cutoff	iFR/QFR cutoff
*Instantaneous flow ratio (iFR)*					
Park et al. [16]	2013	239	Multicenter, prospective	0.80	0.90
RESOLVE [17]	2014	1593	Multicenter, retrospective	0.80	0.90
ADVISE in-practice [18]	2014	392	Multicenter, prospective	0.80	0.90
ADVISE II [19]	2015	690	Multicenter, prospective	0.80	0.89
Fede et al. [51]	2015	89	Single-center, prospective	0.80	0.89
Härle et al. [52]	2015	151	Single-center, prospective	0.80	0.896
Indolfi et al. [26]	2015	123	Single-center, prospective	0.80	0.92
VERIFY 2 [27]	2016	257	Single-center, prospective	0.80	0.90
Kanaji et al. [22]	2016	120	Single-center, prospective	0.80	0.89
IDEAL [20]	2017	366	Multicenter, prospective	0.80	0.89
Ding et al. [53]	2017	229	Single-center, retrospective	0.80	0.86
3V FFR-FRIENDS [21]	2017	975	Multicenter, prospective	0.80	0.90
Scarsini et al. [23]	2017	290	Single-center, prospective	0.80	0.89
Shiode et al. [24]	2017	123	Single-center, prospective	0.80	0.89
Emori et al. [15]	2018	100	Single-center, retrospective	0.80	0.89
Nobre et al. [54]	2018	154	Single-center, prospective	0.80	0.91
Panoulas et al. [28]	2018	46	Single-center, prospective	0.80	0.90
Pisters et al. [55]	2018	515	Single-center, prospective	0.80	0.89
Spagnoli et al. [25]	2018	40	Single-center, prospective	0.80	0.90
*Quantitative flow ratio (QFR)*					
FAVOR Pilot [6]	2016	84	Multicenter, prospective	0.80	0.80
FAVOR II CHINA [7]	2017	330	Multicenter, prospective	0.80	0.80
Yazaki et al. [29]	2017	151	Single-center, retrospective	0.80	0.80
Emori et al. [15]	2018	100	Single-center, retrospective	0.80	0.80
Emori et al. [30]	2018	150	Single-center, retrospective	0.80	0.80
Mejia-Renteria et al. [32]	2018	300	Multicenter, prospective	0.80	0.80
Spitaleri et al. [31]	2018	49	Single-center, prospective	0.80	0.80
FAVOR II Europe-Japan [33]	2018	317	Multicenter, prospective	0.80	0.80
WIFI II [34]	2018	240	Multicenter, prospective	0.80	0.80

**Table 2:** Baseline patient characteristics.

Included studies	Age (y)	Male (%)	Diabetes (%)	Smoking (%)	Hypertension (%)
*Instantaneous flow ratio (iFR)*					
Park et al. [16]	62.8 ± 0.6	161 (68)	66 (28)	64 (27)	133 (56)
RESOLVE^*∗*^ [17]	63.4 ± 10.3	(74.9)	(28.1)	(29.4)	-
ADVISE in-practice [18]	67 ± 11	247 (79)	94 (30)	160 (51)	232 (74)
ADVISE II [19]	63.6 ± 10.8	412 (68.9)	209 (35)	135 (22.6)	471 (78.8)
Fede et al. [51]	67 ± 11	41 (76)	14 (26)	-	44 (81)
Härle et al. [52]	67 ± 11	69 (63.9)	-	-	-
Indolfi et al. [26]	64 ± 9	67 (82)	14 (17)	49 (60)	61 (74)
VERIFY 2 [27]	-	136 (69)	31 (15.7)	48 (24.4)	123 (62.4)
Kanaji et al. [22]	66.6 ± 10.3	94 (79.3)	49 (40.8)	83 (69.2)	80 (63.8)
IDEAL [20]	60.6 ± 9.6	209 (69)	67 (22)	128 (43)	157 (52)
Ding et al. [53]	63.7 ± 9.6	119 (75.3)	29 (18.4)	100 (63.3)	105 (66.5)
3V FFR-FRIENDS [21]	63.8 ± 9.7	303 (77.1)	142 (36.2)	72 (18.4)	248 (63.3)
Scarsini et al. [23]	79.8 ± 9.5	84 (50)	60 (35.7)	114 (68.3)	144 (86.3)
Shiode et al. [24]	70.4 ± 8.7	77 (74.8)	40 (39)	28 (27.1)	82 (80)
Emori et al. [15]	70 ± 10	71 (71)	48 (48)	21 (21)	73 (73)
Nobre et al. [54]	67.3 ± 11	89 (64.5)	39 (28.3)	54 (39.1)	114 (82.6)
Panoulas et al. [28]	63.5 ± 12	35 (56.5)	20 (32.3)	24 (38.7)	40 (64.5)
Pisters et al. [55]	67 ± 10	246 (69)	74 (21)	50 (14)	198 (56)
Spagnoli et al. [25]	66 ± 8	35 (88)	17 (43)	10 (25)	33 (83)
*Quantitative flow ratio (QFR)*					
FAVOR Pilot [6]	65.8 ± 8.9	61 (83.5)	17 (27.4)	-	32 (43.8)
FAVOR II CHINA [7]	61.3 ± 10.4	227 (73.7)	86 (27.9)	87 (28.2)	185 (60.1)
Yazaki et al. [29]	72.5 ± 9.5	100 (70.4)	41 (28.9)	33 (23.2)	101 (71.1)
Emori et al. [15]	70 ± 10	71 (71)	48 (48)	21 (21)	73 (73)
Emori et al. [30]	-	116 (77.3)	70 (46.7)	40 (26.7)	125 (83.3)
Mejia-Renteria et al. [32]	64.2 ± 10.3	188 (76)	94 (38)	56 (23)	164 (66)
Spitaleri et al. [31]	62 ± 11	36 (80)	4 (9)	19 (45)	29 (64)
FAVOR II Europe-Japan [33]	67 ± 10	196 (72)	78 (29)	156 (57)	201 (74)
WIFI II [34]	61 ± 8	116 (67)	18 (10)	101 (59)	121 (70)

^*∗*^The total amount of patients in the RESOLVE study is non-available.

**Table 3:** Baseline angiographic characteristics.

Included studies	Single vessel (%)	Multi-vessel (%)	LAD (%)	LCX (%)	RCA (%)
*Instantaneous flow ratio (iFR)*					
Park et al. [16]	-	-	173 (73)	-	-
RESOLVE^*∗*^ [17]	(46.2)	(53.8)	1003 (63)	271 (17)	319 (20)
ADVISE in-practice [18]	141 (36)	247 (63)	259 (66)	39 (10)	55 (14)
ADVISE II [19]	-	-	380 (54.5)	179 (25.7)	138 (19.9)
Fede et al. [51]	-	-	52 (58)	20 (23)	17 (19)
Härle et al. [52]	75 (69.4)	33 (30.6)	66 (43.7)	20 (13.2)	42 (27.8)
Indolfi et al. [26]	-	18 (15)	-	-	-
VERIFY 2 [27]	-	-	148 (57.6)	37 (14.4)	45 (17.5)
Kanaji et al. [22]	89 (74.2)	31 (25.8)	77 (64.2)	16 (13.3)	27 (22.5)
IDEAL [20]	228 (78)	63 (22)	277 (49)	172 (30)	118 (21)
Ding et al. [53]	-	-	146 (63.8)	25 (10.9)	45 (19.6)
3V FFR-FRIENDS [21]	-	-	343 (35.2)	335 (34.4)	297 (30.5)
Scarsini et al. [23]	-	-	-	-	-
Shiode et al. [24]	-	-	90 (73)	4 (3)	29 (24)
Emori et al. [15]	-	-	63 (63)	23 (23)	14 (14)
Nobre et al. [54]	-	30 (21.7)	-	-	-
Panoulas et al. [28]	-	-	53 (85.5)	2 (3.2)	7 (11.3)
Pisters et al. [55]	-	-	218 (43)	88 (17)	108 (21)
Spagnoli et al. [25]	20 (50)	20 (50)	34 (85)	4 (10)	2 (5)
*Quantitative flow ratio (QFR)*					
FAVOR Pilot [6]	-	-	46 (54.8)	12 (14.3)	19 (22.6)
FAVOR II CHINA [7]	-	-	185 (55.7)	49 (14.8)	87 (26.2)
Yazaki et al. [29]	-	-	96 (63.6)	25 (16.6)	26 (17.2)
Emori et al. [15]	-	-	63 (63)	23 (23)	14 (14)
Emori et al. [30]	-	-	97 (64.7)	17 (11.3)	36 (24.0)
Mejia-Renteria et al. [32]	-	-	177 (59.0)	37 (12.3)	49 (16.3)
Spitaleri et al. [31]	-	45 (100)	-	-	-
FAVOR II Europe-Japan [33]	-	-	160 (50)	50 (16)	68 (22)
WIFI II [34]	-	81 (32)	129 (51)	29 (11)	46 (18)

LAD, left anterior descending artery; LCX, left circumflex artery; RCA, right coronary artery.

^*∗*^The total amount of patients in the RESOLVE study is non-available.

## Data Availability

The data used to support the findings of this study are included within the article.

## Supplementary Materials

Supplementary Materials (1) Search strategy for PubMed, Embase, and CENTRAL. (2) Table S1: inclusion and exclusion criteria of included studies. (3) Figure S1: methodological quality of included studies for instantaneous wave-free ratio. (4) Figure S2: methodological quality of included studies for quantitative flow ratio. (5) Figure S3: Deek's funnel plot for iFR studies. (6) Figure S4: Deek's funnel plot for QFR studies.
