# Supplementary material for: Meta-Analysis of Diagnostic Performance of Instantaneous Wave-Free Ratio versus Quantitative Flow Ratio for Detecting the Functional Significance of Coronary Stenosis
Source: Biomed Res Int. 2019 Apr 18;2019:5828931. doi: 10.1155/2019/5828931 (PMC6500690; doi:10.1155/2019/5828931)
Supplement: Supplementary Materials — (1) Search strategy for PubMed, Embase, and CENTRAL. (2) Table S1: inclusion and exclusion criteria of included studies. (3) Figure S1: methodological quality of included studies for instantaneous wave-free ratio. (4) Figure S2: methodological quality of included studies for quantitative flow ratio. (5) Figure S3: Deek's funnel plot for iFR studies. (6) Figure S4: Deek's funnel plot for QFR studies. [file 5828931.f1.docx]

**Supplementary materials**

**Search strategy for PubMed, Embase and CENTRAL**

**1) iFR studies**

PubMed: 141

((((instantaneous wave-free ratio[Title/Abstract]) OR iFR[Title/Abstract])) AND ((("Fractional Flow Reserve, Myocardial"[Mesh]) OR FFR[Title/Abstract]) OR fractional flow reserve[Title/Abstract]))

Embase: 90

No. Query Results Results Date

#8. #5 AND #6 AND ([article]/lim OR [article in press]/lim) 90 10 Jul 2018

#7. #5 AND #6 355 10 Jul 2018

#6. #3 OR #4 744 10 Jul 2018

#5. #1 OR #2 6,326 10 Jul 2018

#4. 'instantaneous wave-free ratio':ab,ti OR 'ifr':ab,ti 717 10 Jul 2018

#3. 'instantaneous wave free ratio'/exp 99 10 Jul 2018

#2. 'fractional flow reserve':ab,ti OR 'ffr':ab,ti 5,184 10 Jul 2018

#1. 'fractional flow reserve'/exp 4,640 10 Jul 2018

CENTRAL: 17

#1 MeSH descriptor: [Fractional Flow Reserve, Myocardial] explode all trees

#2 fractional flow reserve:ti,ab,kw or FFR:ti,ab,kw (Word variations have been searched)

#3 #1 or #2

#4 instantaneous wave-free ratio:ti,ab,kw or iFR:ti,ab,kw (Word variations have been searched)

#5 #3 and #4

**2) QFR studies**

PubMed: 11

((((FFR[Title/Abstract]) OR fractional flow reserve[Title/Abstract]) OR "Fractional Flow Reserve, Myocardial"[Mesh])) AND ((QFR[Title/Abstract]) OR quantitative flow ratio[Title/Abstract])

Embase: 31

No. Query Results Results Date

#5. #1 AND #4 31 10 Jul 2018

#4. #2 OR #3 6,326 10 Jul 2018

#3. 'fractional flow reserve':ab,ti OR 'ffr':ab,ti 5,184 10 Jul 2018

#2. 'fractional flow reserve'/exp 4,640 10 Jul 2018

#1. 'quantitative flow ratio':ab,ti OR 'qfr':ab,ti 82 10 Jul 2018

CENTRAL: 17

#1 MeSH descriptor: [Fractional Flow Reserve, Myocardial] explode all trees

#2 fractional flow reserve:ti,ab,kw or FFR:ti,ab,kw (Word variations have been searched)

#3 #1 or #2

#4 quantitative flow ratio:ti,ab,kw or QFR:ti,ab,kw (Word variations have been searched)

#5 #3 and #4

**Table S1** Inclusion and exclusion criteria of studies

| Study (First author) | Inclusion criteria | Exclusion criteria |
| --- | --- | --- |
| **Instantaneous flow ratio (iFR)** | | |
| Park | Intermediate lesions with FFR measurement | In-stent restenosis, STEMI, CTO lesions, collateral feeders, regional wall motion abnormalities of a target vessel segment, LVEF <40%, primary myocardial or valvular disease, contraindication to adenosine administration, or angiographically visible thrombus at a target lesion. |
| RESOLVE | Patients with stable angina, unstable angina, or NSTEMI undergoing coronary angiography with or without PCI in whom FFR of a single stenosis in a major epicardial coronary artery was performed | Left main disease, heart failure as defined by NYHA class III or IV, respiratory failure requiring intubation or supplementary oxygen, cardiogenic shock, significant arrhythmia precluding waveform analysis and tachycardia with a heart rate >120 beats/min. |
| ADVISE in-practice | Patients required functional intracoronary assessment | Previous CABG, contraindication to adenosine administration |
| ADVISE Ⅱ | Patients required functional intracoronary assessment | Significant valvular pathology, previous CABG, contraindication to adenosine administration, increased troponin, weight > 200 kg, LBBB, severe vessel tortuosity or calcification, LVEF <30% |
| Fede | Patients with at least one intermediate coronary lesion | Patients with STEMI or hemodynamic unstable conditions |
| Härle | Patients with intermediate coronary stenosis and planned invasive functional assessment | Contraindication to adenosine administration |
| Indolfi | Multivessel disease with at least one intermediate stenosis of a major non-culprit epicardial coronary artery | Non-cardiac life-threatening disease, requiring valvular surgery, cardiologist decided not to perform FFR to guide the treatment, hemodynamic instability, ongoing arrhythmias, valve disease, contraindication to adenosine administration |
| VERIFY 2 | Patients with intermediate coronary stenosis requiring FFR measurement | Severe calcific coronary disease, severe tortuosity rendering pressure wire studies difficult or impossible, recent  myocardial infarction within the previous 72 hours, ongoing unstable chest pain, known intolerance of adenosine, or severe asthma |
| Kanaji | Patients with intermediate lesions | A history of CABG, extremely tortuous coronary arteries, severely calcified arteries, ACS, AMI, occluded coronary arteries, left main disease, coronary ostial stenosis, CHF, significant arrhythmia, renal insufficiency or contraindication to adenosine |
| IDEAL | Patients with CAD undergoing physiological lesion assessment | Severe valvular heart disease, AMI with 48h, previous CABG, vessels with myocardial bridging or collateral arteries, and vessels with a previous myocardial infarction |
| Ding | Patients who had pressure-wire studies | Previous CABG |
| 3V FFR-FRIENDS | Patients with stenosis in all 3-epicardial coronary arteries and FFR measurement | LVEF <35%, STEMI with 72h, previous CABG, chronic renal disease, abnormal epicardial coronary flow or planned CABG after diagnostic angiography |
| Scarsini | Patients with severe AS or stable CAD | <70 years, prior myocardial infarction, prior cardiac surgery, PCI with 12 months, tandem lesions in the same coronary artery, contraindication to adenosine administration, acute presentation of CAD, CHF, or LVEF <45% |
| Shiode | Patients with coronary stenosis required functional intracoronary assessment | None |
| Emori | Patients who underwent angiography, iFR and FFR | Incomplete coronary angiography, ostial lesions in coronary arteries, collateral donor arteries, CABG, chronic atrial fibrillation |
| Nobre | Patients with coronary stenosis required functional intracoronary assessment | Patients enrolled in clinical trials involving FFR or iFR |
| Panoulas | Patients with coronary stenosis required functional intracoronary assessment | Patients with inconclusive DSE imaging, previous CABG, multivessel disease, full thickness infarct of the culprit artery, sequential lesions, diffuse disease or significant valvular disease |
| Pisters | Patients with intermediate coronary stenosis measured by physiological | None |
| Spagnoli | Patients with coronary artery stenosis associated with an intermediate iFR (≥0.86 and ≤0.93) | STEMI, functional test on a culprit lesion in NSTEMI patients, hemodynamically unstable patients, uncompensated heart failure and risk of contrast induced nephropathy |
| **Quantitative flow ratio (QFR)** | | |
| FAVOR Pilot | Patients ≥18 years of age with stable angina and indication for invasive coronary angiography and FFR assessment | Contraindications to adenosine or adenosine triphosphate administration, ostial left main or ostial right coronary artery lesion and prior CABG of the interrogated vessels |
| FAVOR Ⅱ CHINA | Adults with suspected or known CAD who were admitted for coronary angiography | MI within 72 h of coronary angiography, severe heart failure, allergy to the contrast agent or adenosine, serum creatinine >150 μmol/l or glomerular filtration rate <45 ml/kg/1.73 m^2^, ineligible for diagnostic intervention or FFR, had factors that might affect angiographic image quality |
| Yazaki | Patients who underwent coronary angiography and FFR measurements | Lack of 2 optimal angiographic projections at least 25° apart, overlapping interrogated vessels with too much shortening without preferred references in proximal or distal vessels, insufficient injected contrast for QCA analysis, and location of the target lesion at the ostium of the left or right coronary artery |
| Emori (Coron Artery Dis) | Patients who underwent angiography, iFR and FFR | Incomplete coronary angiography, ostial lesions in coronary arteries, collateral donor arteries, CABG, chronic atrial fibrillation |
| Emori (Circ J) | Consecutive series of prior-AMI-related and non-related coronary arteries | Incomplete angiographic results, ostial lesions, collateral donor arteries, CABG, chronic atrial fibrillation, prior-MI in non-target coronary arteries |
| Mejia-Renteria | Patients with CAD who underwent comprehensive physiological assessment with FFR and IMR | Ostial left main and ostial right coronary artery target lesions, previous CABG in target vessel, poor angiography image quality, too much overlap or sever tortuosity of target senoses, and no availability of the raw intracoronary physiology studies |
| Spitaleri | Patients ≥18 years old who presented with STEMI within 12 hours after symptom onset and non-infarct related stenosis ≥50% | None |
| FAVOR Ⅱ Europe-Japan | Patients with stable angina or NSTEMI undergoing coronary angiography and FFR measurement | AMI within 72 hours, severe asthma or severe COPD, allergy to contrast media or adenosine, and atrial fibrillation |
| WIFI Ⅱ | Patients with stenosis referred for invasive coronary angiography | <2 projections with visible stenosis, stenosis in the ostium of the right coronary artery or left main coronary artery, no administration of intracoronary nitrates, pressure wire position not documented angiographically |

CABG: coronary artery bypass graft, FFR: fractional flow reserve, AMI: acute myocardial infarction, STEMI: ST-segment elevation myocardial infarction, CTO: chronic total occlusion, LVEF: left ventricle ejection fraction, NSTEMI: non-ST-segment elevation myocardial infarction, PCI: percutaneous coronary intervention, NYHA: New York Heart Association, LBBB: left bundle branch block, ACS: acute coronary syndrome, CHF: congestive heart failure, CAD: coronary artery disease, AS: aortic stenosis, DSE: dobutamine stress echocardiography, QCA: quantitative coronary angiography, IMR: index of microcirculatory resistance, COPD: chronic obstructive pulmonary disease.


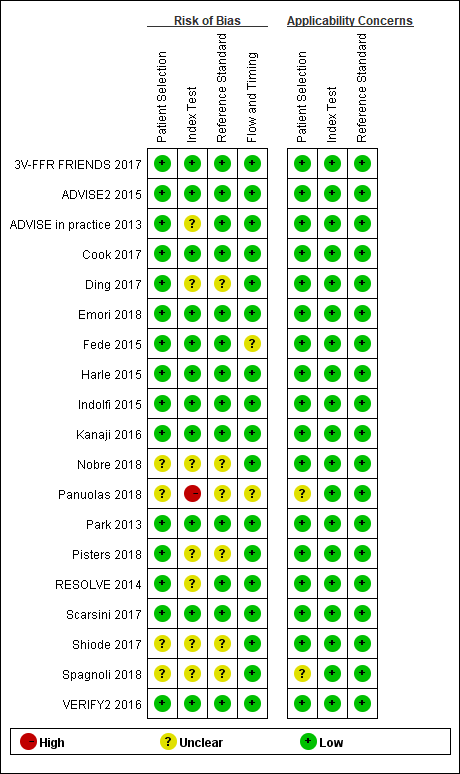


**Figure S1** Methodological quality of included studies of instantaneous wave-free ratio


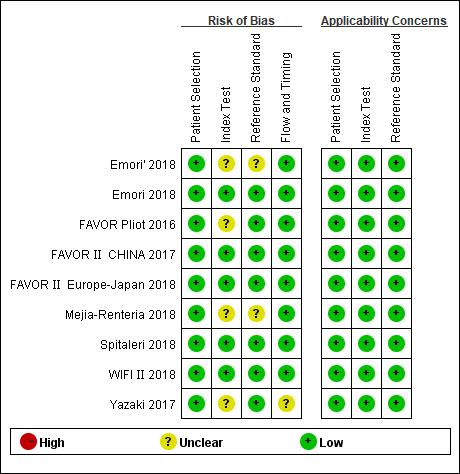


**Figure S2** Methodological quality of included studies of quantitative flow ratio


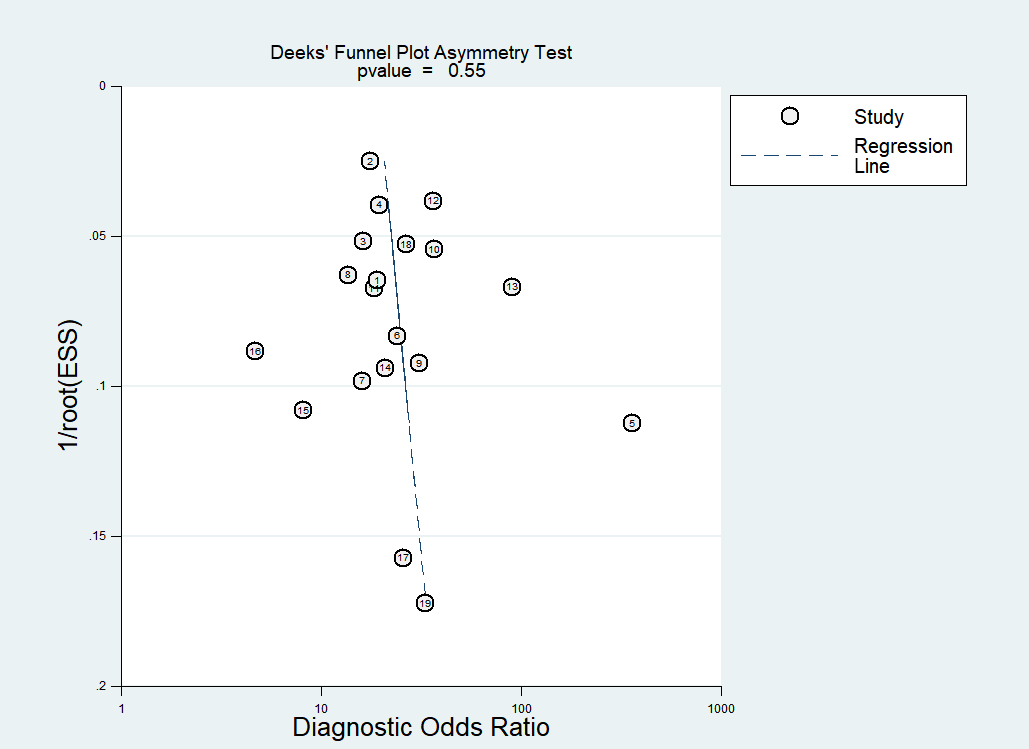


**Figure S3** Deek’s funnel plot for iFR studies


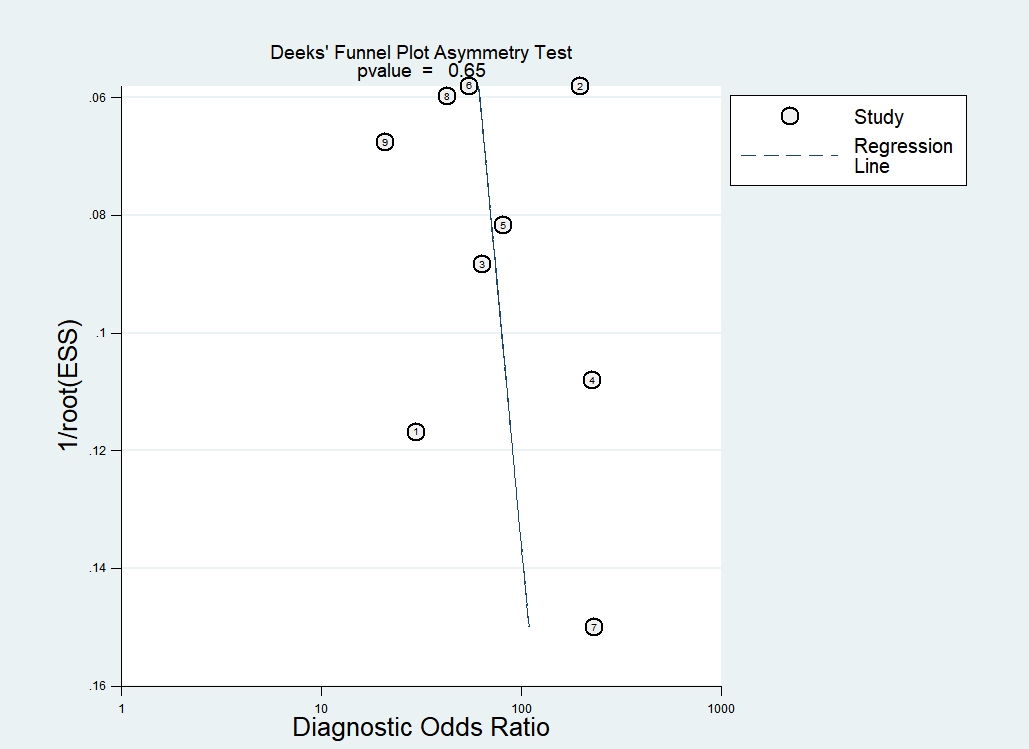


**Figure S4** Deek’s funnel plot for QFR studies
